# Stretchable Supercapacitors Based on Carbon Nanotubes-Deposited Rubber Polymer Nanofibers Electrodes with High Tolerance against Strain

**DOI:** 10.3390/nano8070541

**Published:** 2018-07-18

**Authors:** Juyeon Yoon, Joonhyung Lee, Jaehyun Hur

**Affiliations:** 1 Department of Chemical and Biological Engineering, Gachon University, Seongnam, Gyeonggi 13120, Korea; yjy9474@naver.com; 2 Device & System Research Center, Samsung Electronics, Suwon, Gyeonggi 443-803, Korea

**Keywords:** supercapacitors, stretchable, SBS nanofiber, carbon nanotube, electrospinning

## Abstract

We report a new fabrication method for a fully stretchable supercapacitor based on single wall carbon nanotube (SWCNT)-coated electrospun rubber nanofibers as stretchable supercapacitor electrodes. The deposition conditions of SWCNT on hydrophobic rubber nanofibers are experimentally optimized to induce a uniform coating of SWCNT. For surfactant-assisted coating of SWCNT, both water contact angle and sheet resistance were lower compared to the cases with other surface treatment methods, indicating a more effective coating approach. The excellent electromechanical properties of this electrode under stretching conditions are demonstrated by the measurement of Young’s modulus and normalized sheet resistance. The superb tolerance of the electrode with respect to stretching is the result of (i) high aspect ratios of both nanofiber templates and the SWCNT conductors, (ii) the highly elastic nature of rubbery nanofibers, and (iii) the strong adherence of SWCNT-coated nanofibers on the elastic ecoflex substrate. Electrochemical and electromechanical measurements on stretchable supercapacitor devices reveal that the volumetric capacitance (15.2 F cm^−3^ at 0.021 A cm^−3^) of the unstretched state is maintained for strains of up to 40%. At this level of strain, the capacitance after 1,000 charge/discharge cycles was not significantly reduced. The high stability of our stretchable device suggests potential future applications in various types of wearable energy storage devices.

## 1. Introduction

Stretchable electronics are a class of platform technologies employed in many multidisciplinary fields, such as wearable devices, skin sensors, and bio-integrated devices [1,2]. They generally refer to elastic devices that can sustain their functionality while mechanical stretching is applied. Recently, various studies have been conducted on stretchable electronics, including interconnect electrodes [3], organic light emitting diodes (OLEDs) [4], pressure and strain sensors [5,6], field effect transistors [7,8,9,10], solar cells [11,12], lithium ion batteries [13,14], temperature sensors [15,16], electronic eyes [17], and electrochemical devices [18,19,20,21,22,23,24,25,26,27]. Among various energy-source devices, stretchable supercapacitors have emerged as one of the most indispensable components of stretchable electronics because of the high power delivered compared with other energy devices. Furthermore, a relatively simple fabrication process is another advantage in realizing such devices.

Despite these advantages, there are still some critical issues that have to be overcome to develop high-performance and mechanically durable stretchable supercapacitors. First, the structure of the electrode material, as well as the substrate, should be highly tolerant of strain. In resolving these issues, many different structures have been proposed, including wavy electrode materials on polymeric film substrates, coating active materials on textiles, and wrapping conductive materials on elastic polymeric fibers [28,29,30,31]. Second, the mechanical robustness and stability of other device components (separator and cell holder) upon stretching should also be guaranteed. Third, the fabrication of individual components and the assembly process should be simple. Without satisfying these requirements, stretchable supercapacitors cannot operate properly under harsh mechanical conditions such as stretching deformation.

To date, a variety of different methods have been demonstrated to fabricate stretchable supercapacitors. Single-walled carbon nanotubes (SWCNT), or graphenes deposited onto a buckled rubber substrate, have been used as electrodes [28,32,33]. The coating of conductive ink such as SWCNT or polypyrrole on textile substrate is another approach [29]. Aboutalebi et al. fabricated graphene yarn electrodes that were employed to construct a stretchable energy storage device [34]. Similarly, elastic fibers coated with multiple layers of carbon nanotubes (CNTs) and electrolytes were demonstrated to be another type of stretchable supercapacitor [31]. Furthermore, a two-dimensional, stretchable micro-supercapacitor which used a SWCNT pattern with ion-gel-type or horizontally aligned CNT and a polymeric-gel-type electrolyte on a transparent rubber substrate [35,36]. Wang et al. demonstrated stretchable supercapacitors based on an acrylate rubber/MWCNT composite-film-supported conducting polymer with excellent performance (20.2 F cm^−3^ at 150% strain after 500 cycles of stretching) [37]. In spite of all of these studies, most of these approaches are limited by difficult fabrication processes or poor electromechanical performance, with the latter starting to degrade at relatively low strain. On the other hand, a hybrid nanobuilding block (so called hybrid organic-inorganic materials), i.e., that combines the properties of both organic and inorganic components such as hybrid metal organic frameworks, could be a new alternative to improve the performance of stretchable energy devices provided that these new materials demonstrate both electrical conductivity and high elasticity [38,39,40,41].

Herein, we developed a highly stretchable supercapacitor based on SWCNT-deposited, electrospun (e-spun) elastic rubber nanofiberous mats. Poly(styrene-b-butadiene-b-styrene) (SBS) was used as an e-spun nanofiber because of its great elasticity, while a highly stretchable silicone gel mixture (Ecoflex) was adopted as a substrate. Ecoflex has a Young’s modulus of ~60 kPa, far softer than that (~1.5 MPa) of commonly used polydimethylsiloxane (PDMS), affording much improved mechanical properties under stretching [42]. The SWCNTs were deposited onto e-spun SBS via a simple dipping and drying processes. Because this coating process was highly influenced by the surface properties of the SBS nanofibers, the most appropriate surface treatment was determined experimentally. A bare e-spun SBS nanofiberous mat immersed in ionic liquid was used as an elastic separator. Supercapacitors produced using our approache are fully stretchable because all constituent components are durable and functional under stretching. The main advantages of our approache are a remarkably simple fabrication process without the need of expensive equipment and excellent performance in terms of capacitance retention during the charge-discharge processes under a stretched state. The facile fabrication and excellent performance of our device meet all the requirements for stretchable supercapacitors mentioned earlier, and thus, present a promising stretchable energy storage device for various applications.

## 2. Experiments

### 2.1. Preparation of Electrospun SBS Nanofibrous Mats

SBS solution (Aldrich Inc., St. Louis, MO, USA, Mw = 140,000 g/mol, styrene = 30 wt.%) was prepared by dissolving SBS (10 wt.%) in a solvent mixture of tetrahydrofuran (THF, Aldrich Inc., St. Louis, MO, USA, 99.9%)/dimethylformamide (DMF, Aldrich Inc., St. Louis, MO, USA, 99.8%) (3:1, *w*:*w*). SBS nanofibers were collected by electrospinning, with SBS solution fed at a rate of 50 μL/min under an applied voltage of 20 kV. Typically, a total volume of 20 mL of SBS solution was electrospun on either octadecyltrichlorosilane (OTS, Aldrich Inc., St. Louis, MO, USA, 90%)-treated Si wafers or disposable wipers (Kimberly-Clark Kimtech Inc, Irving, TX, USA, 20 (l) × 30 (w) cm) attached to a custom-made cylindrical collector rotating at 500 rpm. In preparing the OTS-treated Si surface, Si wafers were first cleaned with piranha solution (H_2_SO_4_ (3):H_2_O_2_ (1), *v*:*v*) at 80°C for 10 min, H_2_SO_4_ (Aldrich Inc., St. Louis, MO, USA, 95–98%), H_2_O_2_ (Aldrich Inc., St. Louis, MO, USA, 30%)) to remove any organic contaminants. The Si wafers (Wanxiang silicon-peak electronics Inc., Zhejiang, China, Si (100)) were then dipped in OTS/toluene solution (0.5 wt.%, toluene (Aldrich Inc., St. Louis, MO, USA, 99.8%)) for 5 min. In this process, the trichlorosilane group of OTS reacted with hydroxyl groups on the Si substrate to form a self-assembled monolayer (SAM). To remove the residual OTS, the OTS-treated Si wafers were sonicated (Nexul, Seoul, Korea, NXPC-3010B, 60 Hz, 300 W) in chloroform for 5 min at room temperature. The electrospun SBS nanofibrous mat was carefully peeled off from either OTS-treated Si wafers or Kimtech (if the thickness of SBS nanofibrous mat was too thin, or too much tension was applied during the peeling process, the SBS nanofibrous mat was torn off). The SBS nanofiber mat was cut into 2.0 cm (l) × 1.2 cm (w) pieces using scissors.

### 2.2. Deposition of SWCNT on SBS Nanofibrous Mats

A SWCNT (Hanwha Nanotech Corp., Seoul, Korea, ASP-100F, 90%, length 10–20 μm, diameter 10–20 nm, 100 S/cm) ink solution was prepared by dispersing 10 mg of as-purchased SWCNTs with 5 mg of sodium dodecylbenzene sulfonate (SDBS, Aldrich Inc., St. Louis, MO, USA) in 10 mL of deionized (DI) water using a probe-type sonicator (950 W, Ningbo SCIENTZ Biotechnology Corp., Zhejiang, China) for 2 h. UV/ozone (UVO) surface treatment, polydopamine coating, and fluorinated surfactant (Aldrich Inc., St. Louis, MO, USA, Zonyl FS-300) treatment were carried out to make the SBS nanofibrous mats hydrophilic. For the UVO treatment, SBS nanofibrous mats were placed in a UVO chamber (Novasan Inc., Boone, IA, USA, Jelight 144AX) for 15 min. For the polydopamine coating, the SBS nanofibrous mats were dipped in the dopamine (Aldrich) solution (2 mg/mL of dopamine dissolved in 10 mM Tris-HCl (Aldrich Inc., St. Louis, MO, USA, pH 8.5)) for 12 h at room temperature. The color of the SBS nanofibrous mats changed from white to dark brown, indicating the formation of polydopamine on the nanofiber surfaces. Lastly, for the fluorinated surfactant treatment, 0.1 wt.% of Zonyl FS-300 (Aldrich Inc., St. Louis, MO, USA) was applied to the SBS nanofiber mat. Then, the SBS nanofibrous mat was simply dipped into SWCNT/SDBS solution for 3 min, gently washed with DI water several times, and vacuum dried at room temperature.

### 2.3. Assembly of A Supercapacitor Cell

The supercapacitor was assembled as a two-electrode system, with 1-Ethyl-3-methylimidazolium bis(trifluoromethylsulfonyl)imide (C-TRI Corp., Gyeonggi-do, Korea, [EMIM][NTf2]) as the electrolyte. The Ecoflex rubber film (Smooth-on Inc., Macungie, PA, USA, Supersoft 0010) was prepared by mixing rubber and a curing agent (1:1, *v*:*v*), and casting it with a thickness of 1 mm. The cured Ecoflex film was cut to a size of 2.5 cm (l) × 2.0 cm (w). Freestanding SWCNT-coated SBS nanofibrous mats were attached to the Ecoflex film by gentle pressing for complete adhesion. Owing to the highly tacky nature of the Ecoflex surface, the SWCNT-coated SBS nanofiber (SWCNT/SBS) mats attached firmly to the rubber surface and served both as porous electrodes and as current collectors. Separately, SBS nanofibrous mats (2.2 cm (l) × 1.5 cm (w)) immersed in [EMIM][NTf2] were used as separators between pairs of identical Ecoflex/SWCNT-coated SBS nanofibrous mat electrodes. To ensure good electrical contacts, a copper strip was attached to the edge of each SWCNT-coated SBS fiber mat and a clip was placed in the middle of each cell. The assembled supercapacitors were mounted onto a custom-made sample stage for electrochemical and stretching experiments.

### 2.4. Measurement of Electrochemical Performances

Electrochemical measurements of the stretchable supercapacitors were carried out on a custom-made sample stage. Cyclic voltammetry (CV) and galvanostatic charge-discharge (GCD) measurements were performed using a Bio-Logic VSP potentiostat/galvanostat in a voltage window of 0.0 V to 3.0 V, with sweep rates of 5–100 mV s^−1^ for CV and current densities of 0.021–0.208 A cm^−3^ for galvanostatic measurements. For two-electrode configuration, the volumetric capacitance (*C_v_*) was calculated from the CV or GCD discharge curve using the following equation [19]:

(1) $$C_{v}=\int_{a}^{b}\frac{I\left(E\right)}{\upsilon V\left(b-a\right)}dE\;(\mathrm{from}\;\mathrm{CV}\;\mathrm{curve})$$

where *I(E)*, *υ*, *V*, *a*, and *b* refer to the measured current, the scan rate, the volume of the two electrodes, the starting and the ending voltage of the CV measurement, respectively.

(2) $$C_{v}=\frac{2I}{V\left(\frac{\Delta E}{\Delta t}\right)}\;(\mathrm{from}\;\mathrm{GCD}\;\mathrm{curve})$$

where *I*, *V*, Δ*E*, and Δ*t* denote the applied current, the volume of the two electrodes, the applied voltage, and the discharge time after IR drop, respectively. The long-term cycling test was performed at 0.083 A cm^−3^ with a cutoff potential of 3.0 V.

### 2.5. Characterization

The morphology of the SWCNT-coated SBS nanofibers was observed by field-emission scanning electron microscopy (FESEM, JSM 7000F, JEOL Ltd., Tokyo, Japan). The sheet resistance of the SWCNT-coated SBS nanofibrous mats was measured using a four-point probe with a linear probe head (1.0 mm gap, Jandel HM21, UK). The contact angle of a water droplet on the electrode was measured using a drop-shape analysis system (DSA10-MK2, Krüss, Germany). The mechanical property of the electrospun film was measured using a uniaxial tensile test (Lloyd Instruments Ltd., West Sussex, UK).

## 3. Results and Discussion

### 3.1. Preparation of Stretchable Supercapacitors

**Figure 1:**
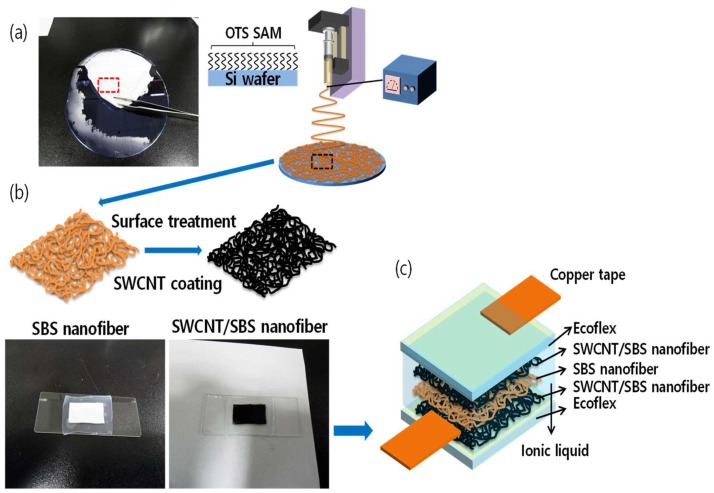
Schematic description of the stretchable supercapacitor fabrication process. (**a**) Electrospinning of SBS on the OTS SAM-treated silicon wafer; (**b**) SWCNT coating on SBS nanofibrous mat/Ecoflex after surface treatment (Photographs of SBS nanofiber mat and SWCNT/SBS stretchable electrode were presented below); (**c**) Assembled stretchable supercapacitor device.

Figure 1 presents a schematic diagram of the methodology for fabricating stretchable supercapacitors. In order to make a nonwoven mat of SBS nanofibers, a sufficient amount of SBS (~2 g) was electrospun on the collector.

Briefly, an SBS solution dissolved in a co-solvent (THF:DMF, 3:1, *w*:*w*) was electrospun on OTS-treated Si wafer. The freestanding film of SBS nanofiber that was peeled off this collector was immersed in an SWCNT/SDBS solution after surface treatment; this was required to change the surface wetting property. The SWCNT-coated SBS nanofiber mat was adhered to the Ecoflex, which acted as a stretchable substrate. Another SBS nanofiber mat immersed in ionic liquid ([EMIM][NTf2]) was used as a stretchable separator and inserted in-between two identical Ecoflex/SWCNT-coated SBS nanofiber mats. Then, a Cu strip was attached to the edge of each electrode for good electrical contact. As more SBS nanofibers accumulated, the mechanical strength (i.e., tolerance with respect to breakage) increased, but the effective elasticity of the freestanding film decreased (Figure S1). Specifically, the rupture strain of 176% for the 4 g SBS nanofiber was much higher (i.e., stronger) than that (126%) of a 1 g SBS nanofiber. On the other hand, a Young’s modulus of 0.77 MPa for the 1 g SBS nanofiber was lower (i.e., more elastic) than that (1.27 MPa) of the 4 g counterpart (Figure S1). The current collectors used in our experiment were either OTS SAM-treated silicon wafers or commercially available disposable wipers. Their highly hydrophobic surface allowed the nonwoven mat of SBS nanofibers to be peeled off easily (Figure 1a) without any damage, and used as freestanding films. The variations in thickness of the detached nanofiber mats were within 10% of the average thickness (typically ~100 μm, Figure S2). SWCNT were coated on the SBS nanofibers using a simple dipping and drying process (Figure 1b). The freestanding SWCNT/SBS films were attached to Ecoflex rubber. The sticky surface of Ecoflex allowed strong adhesion to the films without need for any adhesives or additional binder. An additional freestanding film of SBS nanofiber was used as a stretchable separator. Supercapacitor cells were prepared by sandwiching a SBS nanofiber mat separator immersed in an ionic liquid ([EMIM][NTf2]) between two SWCNT/SBS electrodes attached on Ecoflex substrates (Figure 1c).

### 3.2. Effect of SBS Nanofiber Surface Treatment on the SWCNT Coating

**Figure 2:**
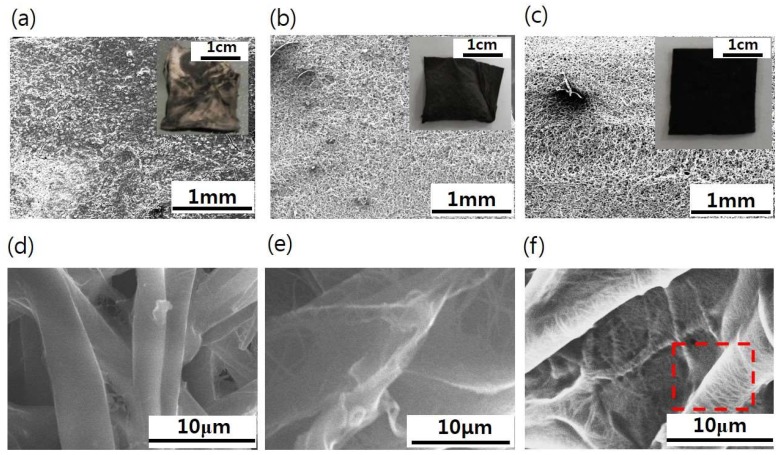
Field-emission scanning electron micrographs (FESEM) of the SWCNT/SBS nanofiber mats following different surface treatments: (**a**,**d**) UVO treatment; (**b**,**e**) Polydopamine treatment; (**c**,**f**) Zonyl treatment. The insets in (**a**–**c**) show photographs of the SWCNT/SBS nanofiber mats. The red dotted box in (**f**) clearly shows the densely coated SWCNT on SBS nanofiber.

The morphologies of the SWCNT-coated SBS nanofibrous mats were examined upon applying different surface treatments to the SBS nanofibrous mats. Normally, aqueous SWCNT solutions dispersed with a surfactant (SDBS) hardly wet the SBS nanofiber surface because of the strong intrinsic hydrophobic property of SBS. Moreover, the rugged surface of the SBS nanofibers further increases its hydrophobicity. Consequently, in order to coat the SWCNTs, the wettability should be improved by an appropriate surface treatment that can increase the surface energy of the SBS nanofibrous mats. Figure 2 compares the morphologies of SWCNT/SBS nanofibrous mats after three different treatments of the SBS nanofiber surface, namely: ultraviolet ozone (UVO), polydopamine coating, and fluorinated surfactant (Zonyl) treatments. It has been established that these are widely used techniques to improve surface wettability, leading to enhanced adhesion. Following UVO treatment, the SWCNT did not uniformly adhere to the SBS nanofiber surface (see Figure 2a,d, and the inset of Figure 2a). 

This is a consequence of the rugged surface of the SBS nanofibrous mats, which does not allow UVO to reach all surfaces within the three-dimensional fibrous matrix. For the polydopamine coating, the nanofiber films were simply immersed in the dopamine solution under basic conditions, resulting in polydopamine formation on the surface of the fibers [43]. However, in this case, although the uniformity of the SWCNT coating was much improved compared with UVO treatment, the resulting SWCNT density was still insufficient, as revealed in Figure 2b,e. Lastly, the nanofiber surfaces were treated with a fluorinated surfactant by coating Zonyl onto SBS nanofibers. Zonyl-treated freestanding SBS nanofiber film was dipped in the SWCNT solution to coat the SWCNT. The change in wetting behavior of SWCNT for Zonyl-treated SBS nanofiber was remarkably different from that of the untreated SBS nanofibrous mat. Indeed, the improvement of coating behavior in the presence of Zonyl is evident in the micrograph shown in Figure 2c,f, where the density of SWCNT on the SBS nanofibers is far greater than those of the UVO (Figure 2d) and polydopamine treatments (Figure 2e). The number of SWCNT layers was generally 1–2 layers on the SBS nanofiber, resulting in a SWCNT thickness of ~10–40 nm, as shown in Figure 2f and Figure S2 (e.g., the diameter of SWCNT is ~10–20 nm).

### 3.3. Wetting, Mechanical and Electrical Characteristics of SWCNT/SBS Nanofibrous Mats Following Different Surface Treatments

**Figure 3:**
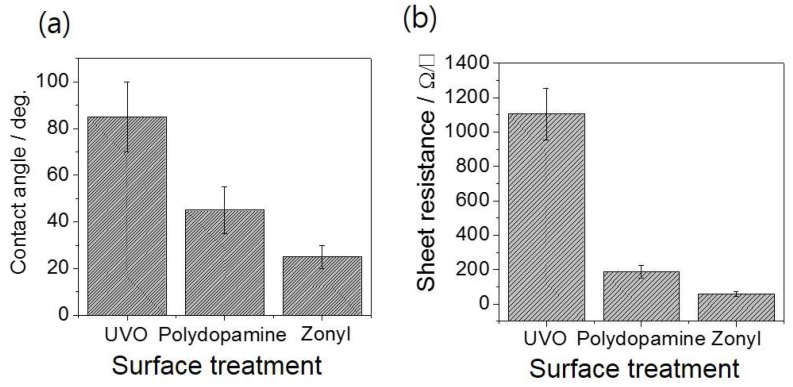
(**a**) Contact angle of the water droplet on the SBS nanofiber film after different surface treatments (UVO, Polydopamine, Zonyl); (**b**) Sheet resistance following three different surface treatments.

The characteristics of SWCNT/SBS nanofibrous mats after the different surface treatments were further quantitatively analyzed by measuring their wetting and electrical properties. Figure 3a,b reveals the contact angle and sheet resistance of SWCNT/SBS after three different treatments (UVO, polydopamine, and Zonly).

The contact angle (25° ± 5°) of the Zonly-treated surface was much smaller than that of other surface treatment methods (85° ± 15° and 45° ± 10° and for UVO and polydopamine treatment, respectively, Figure 3a), which was consistent with the results observed in both photographs and SEM images. From the sheet resistance measurements, they were measured to be 1105 ± 151, 187 ± 38, and 58 ± 15 Ω/□ for the SWCNT/SBS following UVO, polydopamine, and Zonyl treatments, respectively (Figure 3b). Here, it is of note that the thickness and surface area of SBS nanofiber mats were fixed as ~100 μm and 2.2 cm (l) × 1.5 (w) cm, and that the substrate dipping conditions were identical. 

**Figure 4:**
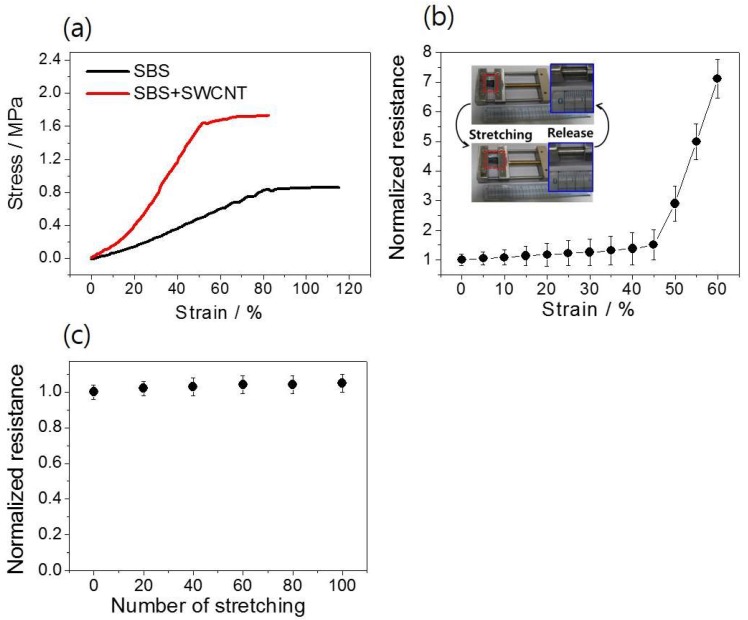
Mechanical and electrical properties of the SWCNT/SBS nanofiber mat electrodes. (**a**) strain-stress curve for SBS and SWCNT-coated SBS nanofiber film; (**b**) Variation of the normalized sheet resistance as a function of the applied strain for Zonyl-treated SWCNT/SBS nanofiber mat electrodes. The inset shows the photograph of the stretching apparatus at different stages of the stretch/release cycle; (**c**) Normalized resistance as a function of the number of stretching cycles at 40% strain.

For the best sample (Zonyl-treated SWCNT/SBS), the mechanical property of the nanofiber film was measured using a uniaxial tensile test (Figure 4a). For the SBS nanofiber film, the Young’s modulus was measured to be ~0.94 MPa. On the other hand, the Young’s modulus of SWCNT-coated SBS nanofiber film was ~4.22 MPa, indicating a much rigid film after SWCNT coating. For both cases, there were strain values at which Young’s modulus suddenly changed; these were ~45% and ~80% of the strain for SBS and SWCNT/SBS, respectively. After these points, the nanofiber films undergo irreversible deformation, thus suggesting the maximum tolerable strain for each case. Figure 4b presents the normalized resistance (η = R/R_o_, where R_o_ and R refer to the sheet resistance of SWCNT/SBS sheet before and after stretching, respectively) as a function of the applied strain. For a stretched SWCNT/SBS, the normalized resistance with respect to the unstretched state remained almost invariant up to ~45% strain, which coincides with our previous tensile test. Beyond this threshold value, the resistance increased radically, implying a breaking of most of the percolation network between the SWCNT. This point is higher than those (~30%) reported in the literature, where a buckled substrate was utilized in stretching experiments [18,25].

**Scheme 1:**
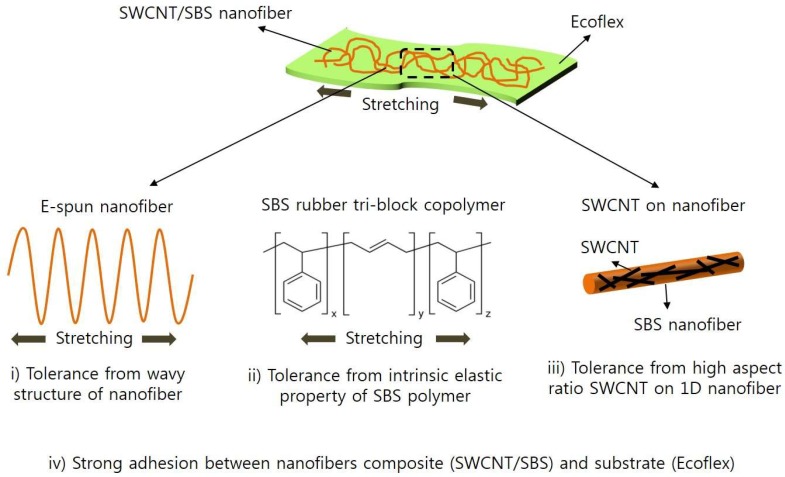
Mechanism of high tolerance against strain for SWCNT/SBS nanofiber/Ecoflex stretchable electrode.

There are four advantages to our approach that contribute to the increased threshold value of the percolation break point. First, the entangled structure of the one-dimensional electrospun SBS nanofibers is already as tolerant to stretching as the previously-used buckled substrates [3,4] (Refer to Scheme 1). In other words, the one-dimensional form factor of the SBS nanofibers that consists of the two-dimensional mat can better sustain elongational strain than its two-dimensional counterpart (i.e., SBS thin films). Indeed, this effect was directly confirmed by the observation of both SBS and SWCNT-SBS nanofibers before and after stretching (Figure S3). It was found that the applied strain induced only alignment of individual fibers, without any damage because of the buckled structure of as-prepared nanofibers.

Second, an intrinsically high elasticity (i.e., Young’s modulus ~0.94 MPa) [44] of electronspun SBS polymer significantly contributes to a high tolerance against mechanical stretching. Third, the percolation network formed by multiple SWCNTs with a high aspect ratio deposited on the nanofiber effectively increases the stain at which the percolation network breakage takes place. Lastly, the strong adhesion between nanofiber composite (SWCNT/SBS) and Ecoflex substrate is another reason for the high endurance of the SWCNT/SBS streatchable electrode. Figure 3c displays the normalized resistance of SWCNT/SBS stretchable electrodes for multiple stretch/release deformations at 40% strain applied repeatedly. The normalized resistance increased less than 5% over the first 100 cycles of stretch/release, suggesting that the three-dimensional SWCNT/SBS porous sheet electrode possesses high mechanical stability against strain. However, when the strain was greater than 50%, the normalized resistance increased irreversibly with the increased number of deformations. This resistance increase with repeated deformations is associated with a gradual rupturing of the percolation network that does not reform between cycles. Consequently, the stretching experiments were carried out mainly below 40% strain. 

### 3.4. Supercapacitor Performances at Different Stretching Ratios

**Figure 5:**
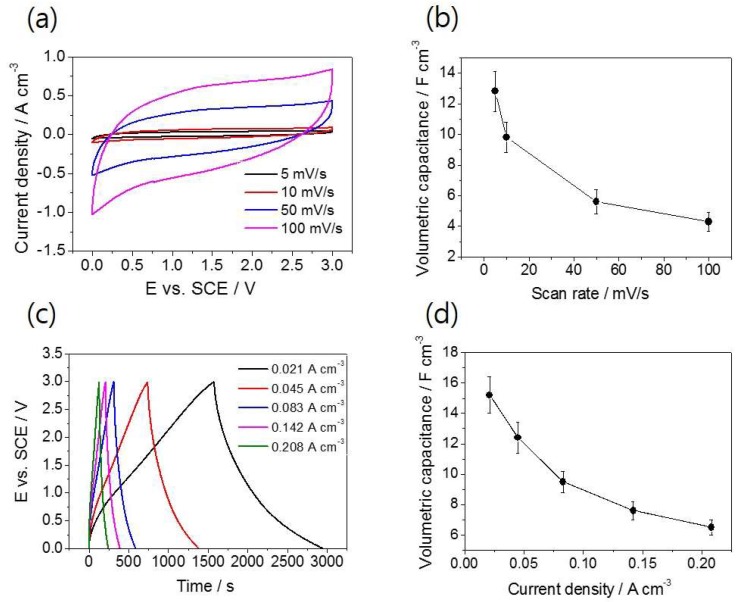
Electrochemical measurements of the as-prepared stretchable supercapacitor. (**a**) Cyclic voltammograms of the supepcapacitor at a scan rate from 0.005 V s^−1^ to 0.1 V s^−1^; (**b**) Volumetric capacitance as a function of scan rate; (**c**) Galvanostatic discharge curves at various current densities; (**d**) Volumetric capacitance as a function of current density.

Prior to the measurement at stretched state, we first evaluated the electrochemical performances of our supercapacitor with a two-electrode configuration in the absence of stretching. Figure 5a displays the CV curves of the devices at scan rates from 0.005 V s^−1^ to 0.1 V s^−1^. All the curves showed the symmetrical and rectangular shape irrespective of the scan rate, indicating that ions diffuse/accumulate effectively in the porous stretchable electrode. The calculated volumetric capacitances were 12.8, 9.8, 5.6, and 4.3 F cm^−3^ at scan rates of 0.005, 0.01, 0.05, and 0.1 V s^−1^ (Figure 5b). Figure 5c shows the GCD curves within a potential window of 3.0 V at current density from 0.021 A cm^−3^ to 0.208 A cm^−3^. Slight deviation from the linear relationship between potential and time was acquired at different current densities (Figure 5c). This behavior is often observed in the symmetric supercapacitors rather than asymmetric counterparts [45,46]; it is reported to be because of the higher internal resistance in symmetric supercapacitors [45,47]. The high IR drop observed in our symmetric device could explain this non-linear behavior. The volumetric capacitance (calculated from the discharge time after IR drop at each current density) varied from 15.2 F cm^−3^ to 6.5 F cm^−3^ within the current densities applied above (Figure 5d).

**Figure 6:**
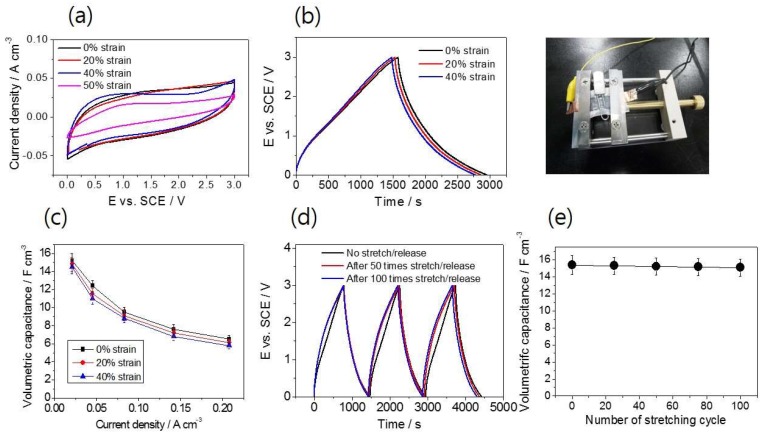
Electrochemical performance of the supercapacitors upon stretching. (**a**) Cyclic voltammograms for devices under 0%, 20%, and 40%, and 50% strain; (**b**) Galvanostatic charge/discharge curves for a devices under 0%, 20%, and 40% strain; and on the right, a photograph taken during a stretching experiment; (**c**) Volumetric capacitance as a function of discharge current density for devices under 0%, 20%, and 40% strain; (**d**) Galvanostatic charge/discharge curves at a current density of 0.045 A cm^−3^ following 0, 50, and 100 times of repeated stretch/release cycles at 40% strain; (**e**) Variation of volumetric capacitance after the different number (0, 25, 75, and 100) of stretch/release cycles.

Figure 6 shows the various electrochemical performances upon applying different stain values. As shown in Figure 6a, with stretching of up to 40% strain, the CV curve approximately maintains its original shape, meaning that no significant irreversible change or damage took place even in the liquid environment inside the device. These behaviors were consistently observed at different voltage scan rates upon 20 and 40% strain (Figure S4). However, at a stretching ratio of 50%, the CV curve began to distort due to the irreversible deformation of the SWCNT/SBS electrodes, as consistent with the results of tensile test and sheet resistance measurements. The volumetric capacitance value (*C_v_*), calculated from GCD measurement (Figure 6b), provided a more quantitative comparison, although the charge-discharge behavior was not perfectly linear (the factors that can influence this non-linear behavior were discussed earlier). For tolerable stretching ratios (less than 40%), the volumetric capacitances at a discharge current density of 0.021 A cm^−3^ were measured to be 15.2, 14.8, and 14.5 F cm^−3^ for stretching ratios of 0%, 20% and 40%, respectively (Figure 6b,c). This suggests that our device is robust even in a highly stretched state. The trend in volumetric capacitance variation as a function of the discharge current density is also similar for unstretched and stretched supercapacitors (Figure 6c).

The decrease in volumetric capacitance with increasing current density observed in both cases is commonly associated with ineffective ion accumulation/dissociation in porous SWCNT/SBS nanofiber mat electrodes. Nevertheless, it was remarkable that the functionality of device remained in good condition. For example, the difference in volumetric capacity between 0% and 40% strain was less than 1%. Figure 6d displays the charge/discharge curves measured after different number (0, 50, and 100) of repeated stretch/release cycles at a current density of 0.045 A cm^−3^ when 40% of strain was applied. Here, it is of note that the first discharge time after IR drop was used to calculate the capacitance for different number of stretching (Figure 6d). The capacitance drops after 50 and 100 times of stretch/release were also less than 1% for both cases (Figure 6e). This small level of performance deterioration after the harsh mechanical deformation clearly highlights a high robustness of our device.

**Figure 7:**
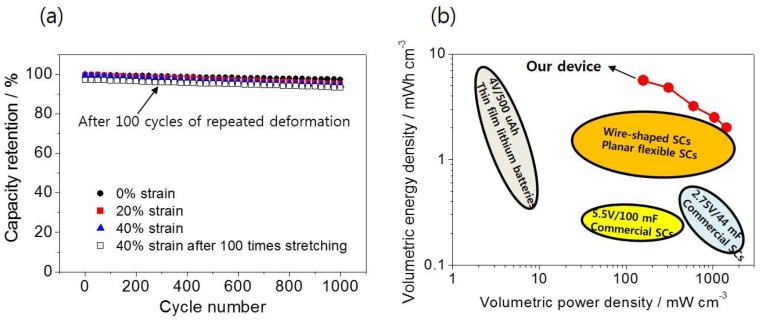
Long-term cycling life (without and with stretching) and Ragone plot. (**a**) Capacitance retention as a function of the number of charge/discharge cycles at a current density of 0.083 A cm^−3^; (**b**) Ragone plots (volumetric energy density vs. volumetric power density) calculated from the galvanostatic charge/discharge curves at various current densities (from 0.021 A cm^−3^ to 0.208 A cm^−3^).

The high mechanical stability of our stretchable device was also confirmed by long-term charge/discharge cycling measurements at a current density of 0.083 A cm^−3^ (Figure 7a). Markedly, the capacitance after 1000 charge-discharge cycles dropped only 2.6%, 4.5%, and 5.5% for 0%, 20%, and 40% strain, respectively. This trivial decrease in capacitance for the stretched state suggests that our device maintains a great level of performance under strains of up to 40%. Even after 100 cycles of stretch/release deformation, although the initial capacity drop was observed compared to as-prepared SWCNT/SBS electrode, the degree of capacity fade (~7%) during 1,000 cycles after 100 cycles of stretch/release was within the similar degree when compared with the case in 0% strain (~3%) without deformation. Additionally, the volumetric power and energy densities in the SWCNT/SBS nanofiber supercapacitors were calculated from the GCD curves (data measured in Figure 5c,d, namely, 15.2, 12.4, 9.5, 7.6, and 6.5 F cm^−3^ without stretching) using the relationships, *E_v_* (mWh cm^−3^) = 0.5*C_v_* Δ*E*^2^ × (1000/3600) and P (mW cm^−3^) = 3600 *E_v_*/*t*, where *C_v_*, Δ*E*, and *t* denote the calculated volumetric capacitance, potential, and time after IR drop, respectively (Figure 7b). A volumetric energy density of 2–5.6 mWh cm^−3^ and a volumetric power density of 158–1450 mW cm^−3^ were obtained for current densities of 0.021–0.208 A cm^−3^ in the SWCNT/SBS nanofibers. Comparing with previous works (Table S1) related to the various stretchable supercapacitors, the performances of our device is excellent, and superior to the characteristics of currently-available various commercial devices (Figure 7b).

## 4. Conclusions

In summary, a new and facile method for fabricating stretchable supercapacitor devices was developed using SWCNT/SBS nanofibrous mats on Ecoflex as stretchable electrodes, SBS nanofiber as a separator, and ionic liquid as an electrolyte. Fluorinated surfactant (Zonyl) treatment allowed the most uniform and effective coating of SWCNT on the SBS nanofibers. Breakage of the percolation network and the conductive pathways in our stretchable electrodes occurred only at ~45% of strain, which was consistently confirmed by mechanical and electrical analyses. Consequently, supercapacitors using our stretchable electrodes maintain their original performance (capacity retention of 93%) even at a highly-applied strain (40%) and after 100 times of repeated stretching/release. The mechanical robustness and excellent electrochemical performances under repeated stretching are associated with the synergetic effects of structural features of constituents (i.e., wavy structure of e-spun rubber nanofibers as well as high aspect ratios of both SWCNT and e-spun fibers), the highly elastic nature (~0.94 MPa) of SBS rubber fibers, and strong adhesion between stretchable SWCNT-SBS fibrous mat and Ecoflex substrate. The proposed fabrication method is simple and versatile compared to conventional approaches because it only requires surface treatment and the dipping process on the nanofiber mat. Furthermore, our method is applicable to any three-dimensional porous substrate, without the need of expensive processes (e.g., evaporation) or the synthesis of complex materials. The stretchable electrode developed herein could be useful not only in wearable energy storage devices, but could also potentially be applicable to various stretchable biosensors and biomedical applications where the electrode is likely to be in frequent displacement (for instance, the patchy-type electrode attached on the skin, elbow, etc.) to monitor the biosignals in human beings. Therefore, our work can present an excellent platform technology for various future stretchable device applications.

## Supplementary Materials

The following are available online at http://www.mdpi.com/2079-4991/8/7/541/s1, Figure S1: Strain-stress curve for SBS nanofiber film with 1 g, 2 g, and 4 g, Figure S2: FESEM image of SWCNT/SBS nanofiber mat with Zonyl treatment, Figure S3: Micrographs of SBS and SWCNT-SBS nanofibers before/after stretching, Figure S4: Cyclic voltammograms of supercapacitors at the scan rate from 0.005 to 0.1 V s^−1^ upon (a) 20%, and (b) 40% strain, title, Table S1: Comparison of the electrochemical performances for various stretchable supercapacitors from the previous researches with our work.
