# Supplementary material for: Stretchable Supercapacitors Based on Carbon Nanotubes-Deposited Rubber Polymer Nanofibers Electrodes with High Tolerance against Strain
Source: Nanomaterials (Basel). 2018 Jul 18;8(7):541. doi: 10.3390/nano8070541 (PMC6071244; doi:10.3390/nano8070541)
Supplement: Supplementary file 1 [file nanomaterials-08-00541-s001.pdf]

Supplementary Materials

Stretchable Supercapacitors Based on Carbon Nanotubes-Deposited Rubber Polymer Nanofibers Electrode with High Tolerance against Strain

Juyeon Yoon[1], Joonhyung Lee[2]*, Jaehyun Hur[1]*

[1]Department of Chemical and Biological Engineering, Gachon University, Seongnam, Gyeonggi 13120, Republic of Korea

[2]Device & System Research Center, Samsung Electronics, Suwon, Gyeonggi 443-803, Republic of Korea

---

*Corresponding author. Tel: +82-31-750-5593; Fax:+82-31-750-8839

E-mail address: joonhyung2009.lee@samsung.com, jhhur@gachon.ac.kr

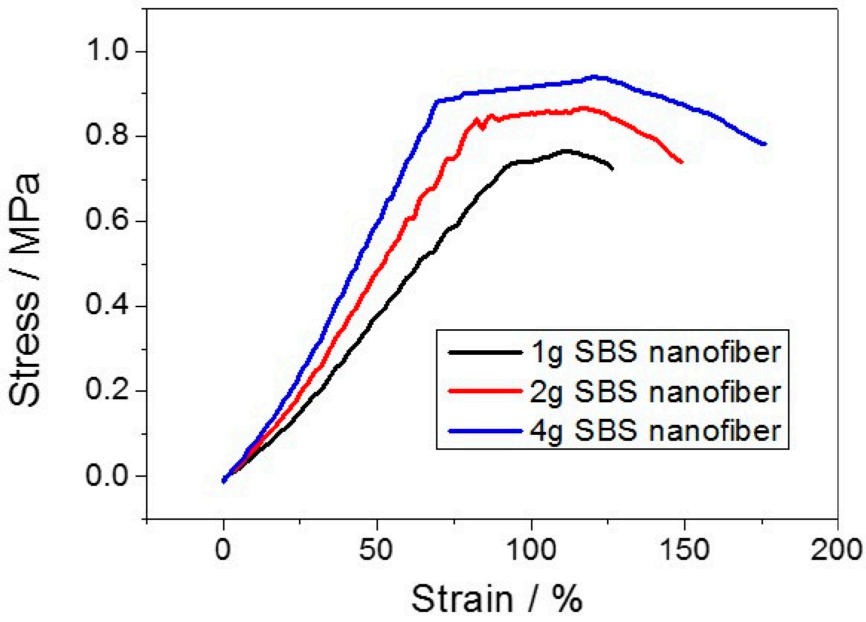

Fig. S1. Strain-stress curve for SBS nanofiber film with 1 g, 2 g, and 4 g.

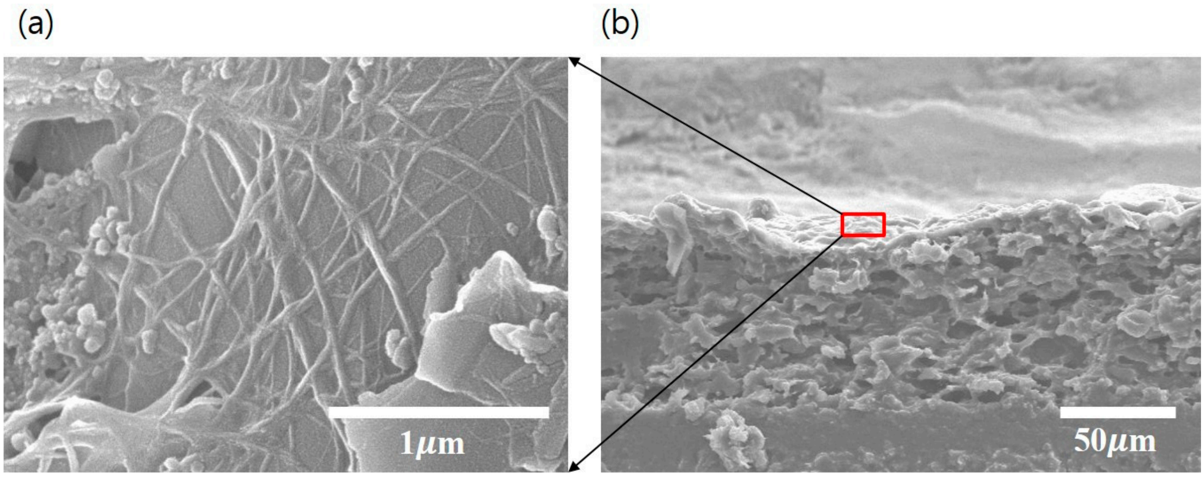

Fig. S2. FESEM image of SWCNT/SBS nanofiber mat with Zonyl treatment: (a) magnified view of coated SWCNT on single SBS nanofiber, (b) cross-sectional view of the film.

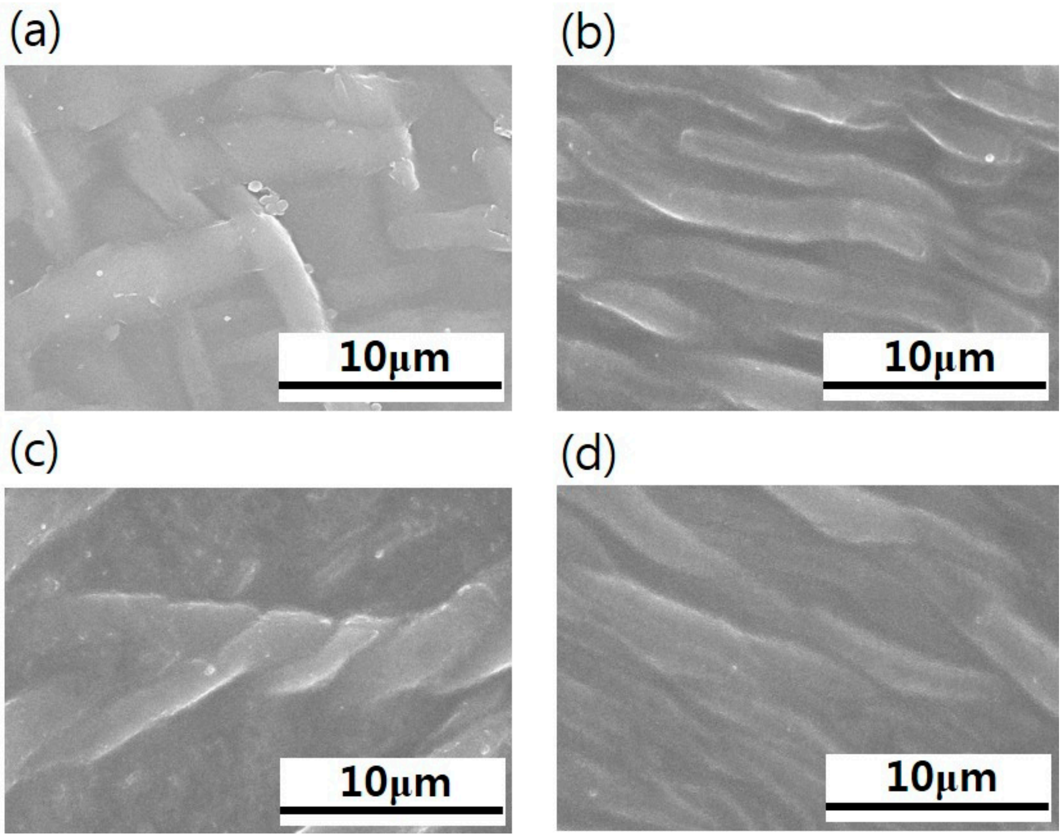

Fig S3. Micrographs of SBS and SWCNT-SBS nanofibers before/after stretching: (a) SBS without stretching, (b) SBS with 40% strain, (c) SWCNT-SBS without stretching, and (d) SWCNT-SBS with 40% strain.

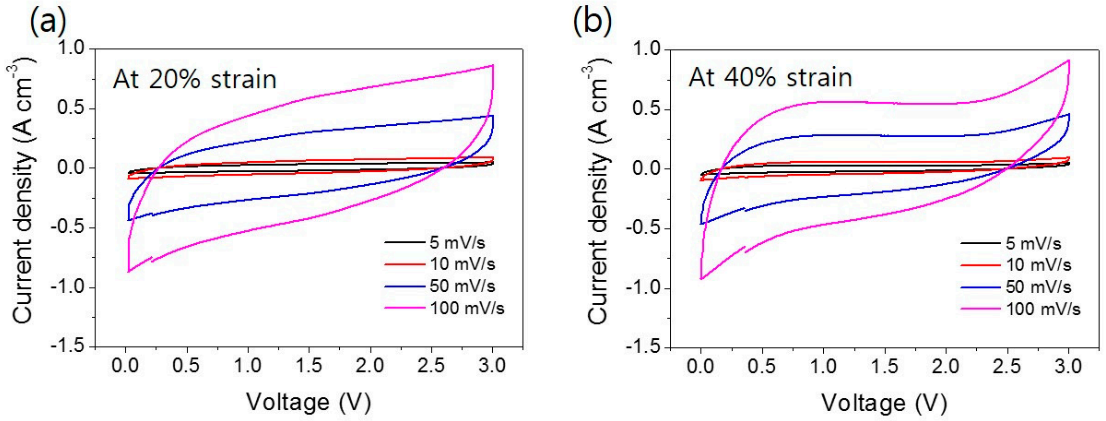

Fig. S4. Cyclic voltammograms of supercapacitors at the scan rate from 0.005 to 0.1 V s^{-1} upon (a) 20%, and (b) 40% strain.

Elec-trode	Stret-ching ratio	Specific capacity (volumetric or areal or gravimetric)	Capacity retention under stretching	Power density (volumetric or gravimetric)	Energy density (volumetric or gravimetric)	Ref.
PPy/rGO/MWCNT	100%	10 F cm^{-3}	82.4%	7.32 mW cm^{-3}	0.36–0.94 mWh cm^{-3}	18
CNT/Ag-coated yarn		5 F cm^{-3}		300–1000 mW cm^{-3}	2 – 5 mWh cm^{-3}	19
PANI@Au@CNT	400%	6 F cm^{-3} (0.2 mF cm^{-2})	95%			20
CNT/MoS_2/PDMS	240%	13 F cm^{-3}				21
Graphene/PDMS	50%	0.65 mF cm^{-2}				22
PANI/CNT	140%	19 F cm^{-3} (72.9 mF cm^{-2})	98.3%	337 mW cm^{-3}	0.7 mWh cm^{-3}	23
3D rGO	130%	75 F cm^{-3} (120 F g^{-1})	97 %			24
CNT@MnO_2/hydrogel	150%	6.6 mF cm^{-2}	90%			25
PPy@CNT	600%		350%			26
Buckled SWNT	131.5%	50 F g^{-1}	94.6%			28
Buckled SWCNT	130%	45 F g^{-1}	92.6%			29
Wrinkled	140%	4.9 uF cm^{-2}				30

graphene		(6.4 F g^{-1})				
CNT/textile	120%	800 mF cm^{-2} (62 F g^{-1})		30 kW kg^{-1}	20 Wh kg^{-1}	31
Acrylate rubber/MWCNT@conductive polymer	150%	20.2 F cm^{-3}			2.14 mWh cm^{-3}	37
SWCNT-coated SBS nanofiber	145%	15.2 F cm^{-3}	93%	158–1450 mW cm^{-3}	2 – 5.6 mWh cm^{-3}	**This work**

Table S1. Comparison of the electrochemical performances for various stretchable supercapacitors from the previous researches with our work.
